# High-Throughput Metabolomics for Identification of Metabolic Pathways and Deciphering the Effect Mechanism of Dioscin on Rectal Cancer From Cell Metabolic Profiles Coupled With Chemometrics Analysis

**DOI:** 10.3389/fphar.2020.00068

**Published:** 2020-02-28

**Authors:** Xin-Xin Wang, Peng-cheng Yu, Jun Li

**Affiliations:** ^1^Heilongjiang Province Land Reclamation Headquarters General Hospital, Heilongjiang Agriculture and Reclamation Bureau, Harbin, China; ^2^College of Traditional Chinese Medicine, Jilin Agricultural University, Changchun, China; ^3^Department of Orthopedics, The Affiliated First Hospital of Heilongjiang University of Chinese Medicine, Harbin, China

**Keywords:** metabolomics, biomarker, natural product, metabolic pathways, mass spectrometry

## Abstract

High-throughput liquid chromatography–mass spectrometry (LC-MS)-based metabolomics can provide the holistic analysis of the low molecular weight endogenous metabolites in cells and reflect the changes of cellular regulation and metabolic pathways. Our study designed to reveal the potentially pharmacological effects of dioscin on SW480 rectal cancer cells using nontargeted metabolomics method to probe into small molecular metabolites and pathway changes. After the cell assay of proliferation, apoptosis, migration, and invasion, the dioscin-treated cell samples were prepared for nontargeted metabolomics analysis based on LC-MS tool to describe the metabolic profiles. Dioscin has prevented cell proliferation and accelerated cell apoptosis, and it also inhibited the SW480 rectal cancer cells’ migration and invasion. A total of 22 metabolites were selected as promising biomarkers of pharmacological reaction of dioscin to rectal cancer, and eight highly correlated pathways including D-glutamine and D-glutamate metabolism, pyruvate metabolism, arachidonic acid metabolism, phenylalanine metabolism, tryptophan metabolism, glycolysis or gluconeogenesis, citrate cycle (TCA cycle), and butanoate metabolism were identified. It showed that strategies based on cell metabolomics are helpful tools to discover the small molecular metabolites to elucidate the action mechanism of drug.

## Introduction

Rectal carcinoma, as the most universal detrimental gastrointestinal lump between the dentate line and the rectosigmoid junction, brings enormous suffering to patients. It becomes the second *dominant reason* for mortality of cancer field in the western countries (Zhang et al., 2013a; Siegel et al., 2014). It is reported that rectal cancer is the third main cause of male and female cancer death in America, and the number of patients with rectal cancer grows by about 1.2 million every year all over the world (Schootman et al., 2017). The etiology of rectal cancer is still not very clear, and the incidence is closely linked to the social environment, eating habits, genetic factors, and others. It is generally accepted that superabundant animal fat and protein intake and inadequate dietary fiber intake are high risk factors for rectal cancer (Vermeer et al., 2014; Zhang et al., 2014a). Because of the low location of the disease, it is easy to be diagnosed by digital rectal examination and sigmoidoscopy (Maas et al., 2015). Currently, operation followed by radiation and chemotherapy is a major therapeutic strategy; however, nearly 30% of invalids with stage II rectal cancer suffer from recurrence and die (Deming et al., 2015). Single target drugs or functional products with less pharmacological activity can’t satisfy the multiple pathological changes. In addition, limited efficiency of therapy also leads to drug resistance and severe toxicity (Chan and Giovannucci, 2010; Panczyk, 2014). It is an imperative need to look for original therapeutic options for the handling of rectal cancer.

With the advancement of modern science and the increase in social needs, more and more experts and scholars pay more attention to multitarget drugs (Zhang et al., 2013b; Zhang et al., 2016a). Owing to the ingredients’ diversity and low toxicity, traditional Chinese medicines (TCMs) have been applied in treating a large number of diseases for a long time and achieved marked multiple-target effects (Zhang et al., 2018). Since the item on artemisinin resisting malaria by Youyou Tu, who has obtained the “2015 Nobel Prize in Hysiology or Medicine”, various kinds of active natural product studies that stem from TCMs have been performed to obtain huge advance (Sun et al., 2018) . Dioscin is a steroidal saponin compound in Dioscoreae rhizoma, which is characterized by antitumor, immunomodulatory, anti-inflammatory, hypolipidemic, anti-AIDS, and other biological activities involved in cell processes, metabolic processes, biological regulation, stress response, signaling pathways (Wang et al., 2014; Zhang et al., 2014b; Zhang et al., 2016c; Zhang et al., 2016d). It also has confirmed that diosgenin produced by dioscin metabolism can exert antitumor effects (Zhang et al., 2013c; Zhang et al., 2018). Hence, dioscin is a hopeful multitarget nominee to fight cancer; however, the underlying mechanisms of dioscin on rectal cancer in metabolic level were little known.

Metabolomics is a high-throughput “omics” technique, which offers holistic identification and quantification measurement of all small endogenous metabolites (MW < 1,500 Da), and is affirmed in resisting carcinoma study (Wang et al., 2013). As a final downstream product *in vivo*, metabolome reflects the changes of enzyme level, cellular regulation, signaling pathway control, genetic variations, and catabolic and anabolic reactions in health and physiopathologic state (Dong et al., 2012). Metabolomics has the ability to simultaneously measure thousands of metabolites to characterize the global and dynamic profiling of disease occurrence and development by distinct association of liquid (LC) and gas (GC) chromatography binded with mass spectrometry (MS) or nuclear magnetic resonance (NMR) (Sun et al., 2013; Zhang et al., 2015a). To date, more valuable information about biomarker findings for forecasting, early diagnosis, accurate curing, and prognosis has been successfully brought about (Wu et al., 2011; Zhang et al., 2014c; Wang et al., 2015; Liu et al., 2018). Metabolomics has its special advantages in the study of complex disease mechanisms and could correlate changes in metabolite content with biological phenotype. In the present study, we explore the potential metabolite biomarkers and pathway for predicting antitumor response of dioscin in SW480 rectal cancer cells and make an attempt to elucidate the protective mechanism of dioscin.

## Experimental Method

### Chemicals and Reagents

Acetonitrile, methanol, and formic acid in HPLC grade were obtained from Merck (Darmstadt, Germany). Pure water for mobile phase and aqueous solution preparation were obtained from a Milli-Q water ultrapure water system (Millipore, Bedford, MA, USA). Leucine enkephalin was bought from Sigma-Aldrich (St. Louis, MO, USA). Dioscin of over 99% purity was bought from Tianjin Chemical Reagent Co. (Tianjin, China). Human SW480 rectal cancer cell lines were from the England collection of cell cultures (Salisbury, UK). Cell culture reagents RPMI1640 and fetal bovine serum (FBS) were gained from China Langchem INC. (St. Caliun, Shanghai). Dimethyl sulfoxide (DMSO) and MTT (3-(4,5-dimethyl-2-thiazolyl)-2,5-diphenyl-2-H-tetrazolium bromide) were purchased from GIBCO (Piscataway, USA). Viacount cell counting reagent and Annexin V-FITC/PI kit were obtained from Sabinsa Corporation (Piscataway, USA). Transwell chamber was obtained from Roche Applied Science (Roche, Germany). Other reagents were of the highest grade commercially accessible.

### Cell Culture

Human rectal cancer cell line SW480 was trained in RPMI-1640 medium (Invitrogen) with 10% fetal bovine serum (FBS). Under an incubator with an atmosphere of 5% CO_2_ at 37°C, SW480 cells were seeded into a 6-wells culture plate at a density of 1.05 × 106 cells per well and then incubated for 24 hours. Then, cold PBS was applied to rinse the cell medium for three times. The growing cells were treated with dioscin for 24, 48, and 72 h, which the stock solution of dioscin (80 c/μ mol·L^−1^) was intended in DMSO and deposited at −20°C. The morphological changes of the growth process of SW480 rectal cancer cell were recorded by inverted optical microscope.

### Cytobiology Analysis

#### IMTT Assay for Cell Proliferation Analysis

SW480 rectal cancer cells were placed in 96-well flat-bottom trays for 24 h common promotion. After the medium was replaced, dioscin was dissolved in DMSO and diluted by the RPMI1640 culture solution at the final concentrations of 5 c/μ mol·L^−1^, 10 c/μ mol·L^−1^, 20 c/μ mol·L^−1^, 40 c/μ mol·L^−1^, 80 c/μ mol·L^−1^ and respectively incubated for 24h, 48h, and 72 h at 5% CO_2_, 37°C. Then, the suspension was discarded and the cells were hatched with DMEM containing 5 mg/ml MTT solution for 3 h. The obtained sediment in each well was dismissed with 150 μl DMSO, and rocked until the crystals dissolved. Control cells were performed with the same bulk of DMSO as the same means. Every group was set six parallel samples. The optical density value (OD) was gauged at 540 nm on a microplate reader abide by computational formula as the following: inhibition rate% = (1−OD treatment group/OD control group) × 100%.

#### Annexin V-FITC/PI Assay for Cell Apoptosis Analysis

According to the kit specification, cell apoptosis of SW480 rectal cancer cells induced by dioscin was determined by the Annexin V fluorescein isothiocyanate (Annexin V-FITC)/propidium iodide (PI) assay. 1.5 × 10^6^/well cells were inoculated in 96-well dishes containing RPMI 1640 solution before dioscin administration for 24, 48, and 72 h at 80 c/μ mol·L^−1^. Then, the harvested cells were reascended in cold PBS solution including 137 mM NaCl, 2.7 mM KCl, 4.3 mM Na_2_HPO_4_-7H_2_O, 1.4 mM KH_2_PO_4_ at pH 7.4 and dyed by Annexin V and PI for 15 min at indoor and dark environment condition. The apoptosis changes and the percentage of apoptosis cells were resolved by FACStar Plus flow cytometer coupled with Flowjo software. The different evident regions of the flow cytometry disperse profile represent cell changing process: lower left region represents movable cells, upper left region represents dead cells, lower right region stands for apoptotic cells in early period, and upper right region stands for apoptotic cells in farther phase. The general apoptosis proportion is the summation of apoptotic cells in the whole process.

#### Transwell Assay for Cell Migration and Invasion Analysis

50 μl Matrigel prediluted with DMEM medium at the ratio of 1 to 8 is placed on the bottom of the chamber surface of the Transwell at 37°C for 6 h. Human SW480 rectal cancer cells treated with DMEM in logarithmic growth phase for 24 h were inoculated in the upper chamber of the Transwell and then treated with different concentrations of dioscin including 10 c/μmol•L^−1^, 20 c/μmol•L^−1^, 40 c/μmol•L^−1^. After 24 h cultivation, the filter was removed, and the uninjured cells on the surface only were removed by a cotton swab. The chamber was set in a 4% volume paraformaldehyde solution for 15 min, and the volume fraction 0.5% crystal violet solution was stained at room temperature for 20 min. Through three times PBS washing and air drying, the number of cells (N) was counted using a light microscope, which the average number expresses the invasive ability of the tumor cells (inhibition inhibition rate = (1 − N experimental group/N control group) × 100%. Compared with the invasive ability experiment, the activity evaluation of migration in human SW480 rectal cancer cells is performed with matrigel at the bottom of the upper chamber of the Transwell culture dish. The remaining procedure is carried out according to cell invasion experiment (Migration inhibition rate = (1 − N experimental group/N control group) × 100%).

### Cell Metabolomics

#### Sample Preparation

Cells from the control group and different dioscin-treated group were prepared for metabolomics study. 1 × 10^6^ cells in each group were disposed by 20 c/μ mol•L^−1^ of dioscin for 24, 48, and 72 h, respectively. In order to obtain parallelism and avoid cell cytotoxicity, cells of the control group were managed by the same dose of DMSO with a proper concentration of less than 0.1%. The culture medium was removed after dioscin treatment, and then cells in the control and therapeutic groups were fleetly washed by 3,000 μl of 37°C deionized water and gathered in 5 ml neat pipe. After being off-center at 1,500 rpm for 15 min at 4°C and eliminating the liquid supernatant, the quenched cells collected in 3 ml centrifuge tube were obtained by 1 ml of cold methanol/water (5:1), and then were performed to ultrasonication with 90 kHz ultrasound frequency and 30 W power on ice bath for 5min. The supernatants of the cell fragment were centrifuged at 12,000 rpm for 15 min at 4°C and then were leached using a 0.22 µm filter membrane for instrumental analysis. Quality control (QC) cell specimen was intended by blending 2 µl each sample.

#### LC–MS Condition

The LC–MS data was fulfilled by an ultrahigh performance liquid chromatography (UPLC) system (1290 series, Agilent Technologies, California, Santa Clara, USA) coupled to high throughput G2Si High-definition mass spectrometry (Agilent 6550, Agilent Technologies, USA) in positive and negative ion modes. Chromatographic separation is achieved in BEH C18 column (2.1 mm × 100 mm, particle size of 1.7 μm) held at 35°C. The optimized gradient mobile phase is composed by A (water: formic acid = 100:0.1) and B (acetonitrile: formic acid = 100:0.1) operated under the linear elution gradient program at 0.8 ml/min flow rate and 2 μl injection volume as follows: 0–1 min, 95% A; 1–2 min, 95–85% A; 2–3 min, 85–70% A; 3–5 min, 70–55% A; 5–8 min, 55–20% A; 8–10 min, 20-0% A, 10–11 min 0–99%, 12–13min 99%. The vintage MS parameters are set as listed below: in positive ion mode ([M + H] ^+^ = 556.2771), the desolvation gas flow of 500 L/h, the capillary voltage of 4,000 V, and the cone voltage of 40 V were set; in negative ion mode ([M − H] ^−^ = 554.2615), the desolvation gas flow of 400 L/h, the capillary voltage of 2,500 kV, and the cone voltage of 30 V were set. The ion source temperature was set at 150°C and the cone gas flow was fixed at 100 L/h in both modes. During data acquisition, all cell samples were randomly injected, and blank samples were prepared with 80% methanol in water, and QC samples were injected every 8 samples. Leucine-enkephalin as an internal standard was applied to supervise the accurate mass measurement and monitor the reproducibility of the system.

### Data Mining and Statistical Analysis

The fresh data of LC/MS from different series were handled though MarkerLynx software (version 4.1, Waters Corporation, MA, USA), which includes retention time (RT), mass range, mass tolerance, intensity threshold, maximum mass, and retention time tolerance. EZinfo 2.0 software is applied for further multivariate data analyses, such as principal component analysis (PCA), orthogonal projection to latent structure-discriminant analysis (OPLS-DA), and variable importance in projection (VIP) score plot. In an unsupervised PCA score plot, metabolic ingredients with higher similarity present the trend of gathering together and the opposite present the trend of separation. OPLS-DA resolution was utilized to comprehend the whole metabolic disorder between untreated cells and dioscin-treated cells, then the S-plot and homologous variable importance in the projection (VIP values) were calculated in the PLS model.

Metabolite peaks with VIP more than 1 and P-value was less than 0.05 in student’s t-test were considered latent biomarkers for more research. A substantiation test was applied to evaluate the legality of the PLS model by contrasting the goodness of fit (R2 and Q2) of the PLS models with the goodness of fit of 200 Y-permutated models. Metabolite identifications were employed by RT, precise MS, MS/MS fragments data, and databases online such as Chempider (http://www.chemspider.com/), HMDB (http://www.hmdb.ca/) as well as KEGG (http://www.genome.jp/kegg/). The pathways and network establishment which are closely associated with dioscin treatment were performed to resolve through MetaboAnalyst 4.0 and ingenuity pathway analysis (IPA) platform for revealing the action mechanism of dioscin on SW480 rectal cancer cells.

## Results

### The Effect of Dioscin on Cell Proliferation and Apoptosis

In **Figure S1A**, the morphology of SW480 rectal cancer cells in the control group was in a good growth state under inverted optical microscope. With the increasing treating time, SW480 rectal cancer cells stimulated by dioscin for 24, 48, and 72 h were evidently changed, which brings out the phenomena of cell growth suppression increase including cell volume shrinkage, lower volume, and bigger cell gap. As shown in **Figure S1B**, when the concentration was less than or equal to 10 *c/μmol·L*^*−1*^, the inhibition rate of the SW480 rectal cancer cells was less than 42%. However, 80 *c/μmol·L*^*−1*^ dioscin in 72 h possessed the highest inhibition rate of 84.5% as the treating time and concentration increased. In **Figures S1C, S1D**, the total apoptosis rate was 68.52 ± 4.03% at a level of 80 *c/μmol·L*^*−1*^ after dioscin treatment, which displayed considerable statistical differences in comparison with the control group (p < 0.01). Similarly, the scale of apoptosis cells in a time-dependent way tended to the right area after 24, 48, and 72 h at 80 *c/μmol·L*^*−1*^ of dioscin in flow cytometry scatter plots. Cells in the control group were centered on the lower left zone suggesting that there was no cell apoptosis. It is implicit that dioscin has latent aptness to hinder SW480 rectal cancer cell proliferation and accelerate cell apoptosis.

### The Effect of Dioscin on Cell Migration and Invasion

In **Figure S2A**, the numbers of migrated cells were decreased with the increase of concentration, which suggested that dioscin inhibited the migration of SW480 rectal cancer cells *in vitro* in a concentration-dependent way. Compared with dioscin in 10 μmol•L^−1^, the number of migrated cells after treatment was significantly reduced in 40 μmol•L^−1^ (P < 0.01). In **Figure S2B**, the invasive ability from 10 μmol•L^−1^ to 40 μmol•L^−1^ was much lower than that of the control group, suggesting that dioscin inhibited the invasive ability of SW480 rectal cancer cells *in vitro*. Compared with dioscin in 10 μmol•L^−1^, the inhibitory ability was increased with increasing concentration, the number of invading cells after treatment was significantly reduced in 40 μmol•L^−1^ (P < 0.01). It showed that dioscin possessed a latent capability to hinder SW480 rectal cancer cells’ migration and invasion.

### Multivariate Statistical Analysis

**Figure 1:**
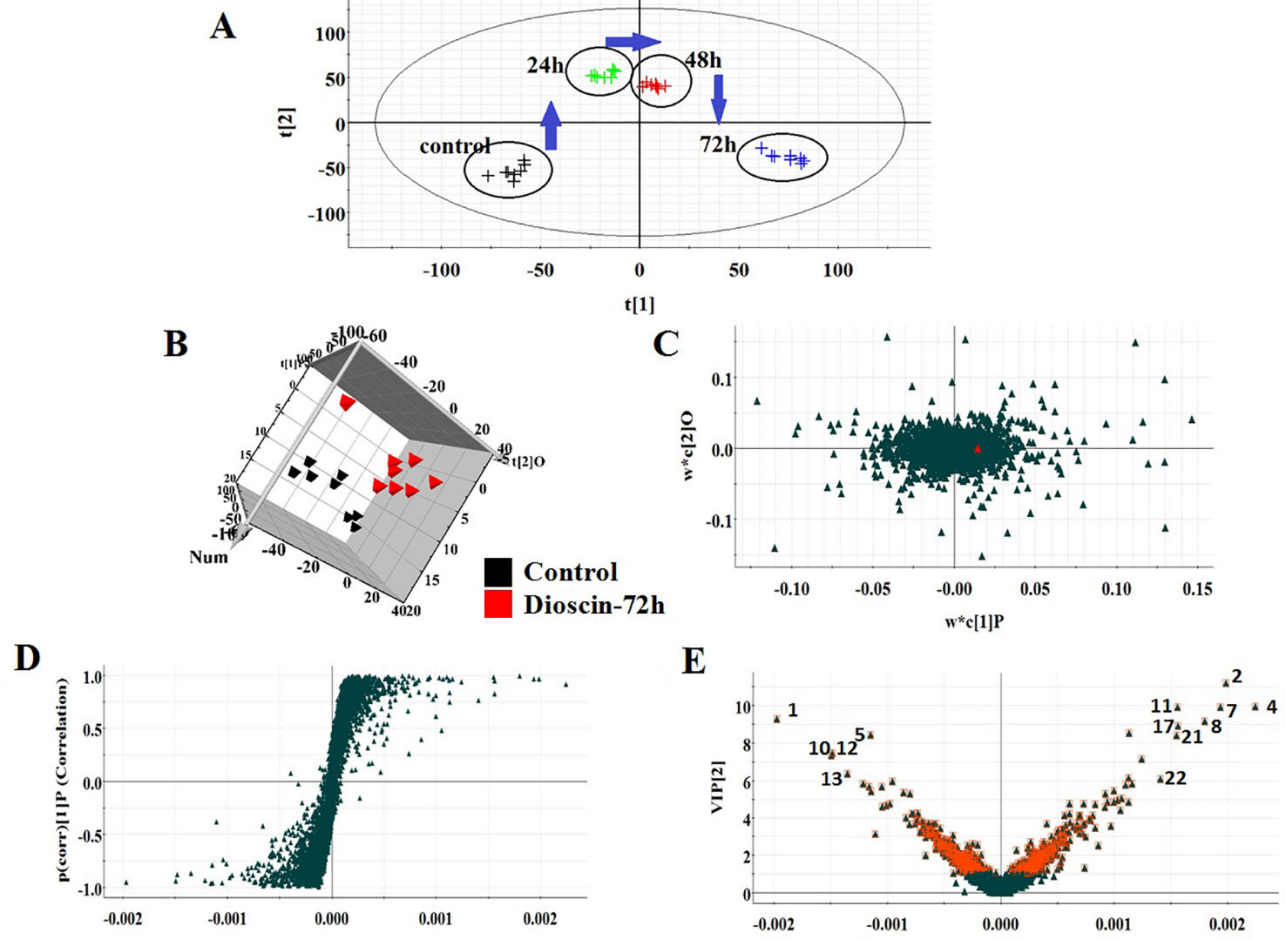
PCA scores’ plot of cell metabolism trajectory in the dioscin-treated group at different time points in positive mode **(A)**. 3D OPLS-DA score plot of cell UPLC-MS spectra data between control and 72 h dioscin-treated group in positive mode **(B)**. Loading-plot of OPLS-DA model of UPLC-MS spectra data between control and 72 h dioscin-treated group in positive mode **(C)**. S-plot of OPLS-DA model of UPLC-MS spectra data between control and 72 h dioscin-treated group in positive mode **(D)**. VIP-plot of OPLS-DA model of UPLC-MS spectra data between control and 72 h dioscin-treated group in positive mode **(E)**.

**Figure 2:**
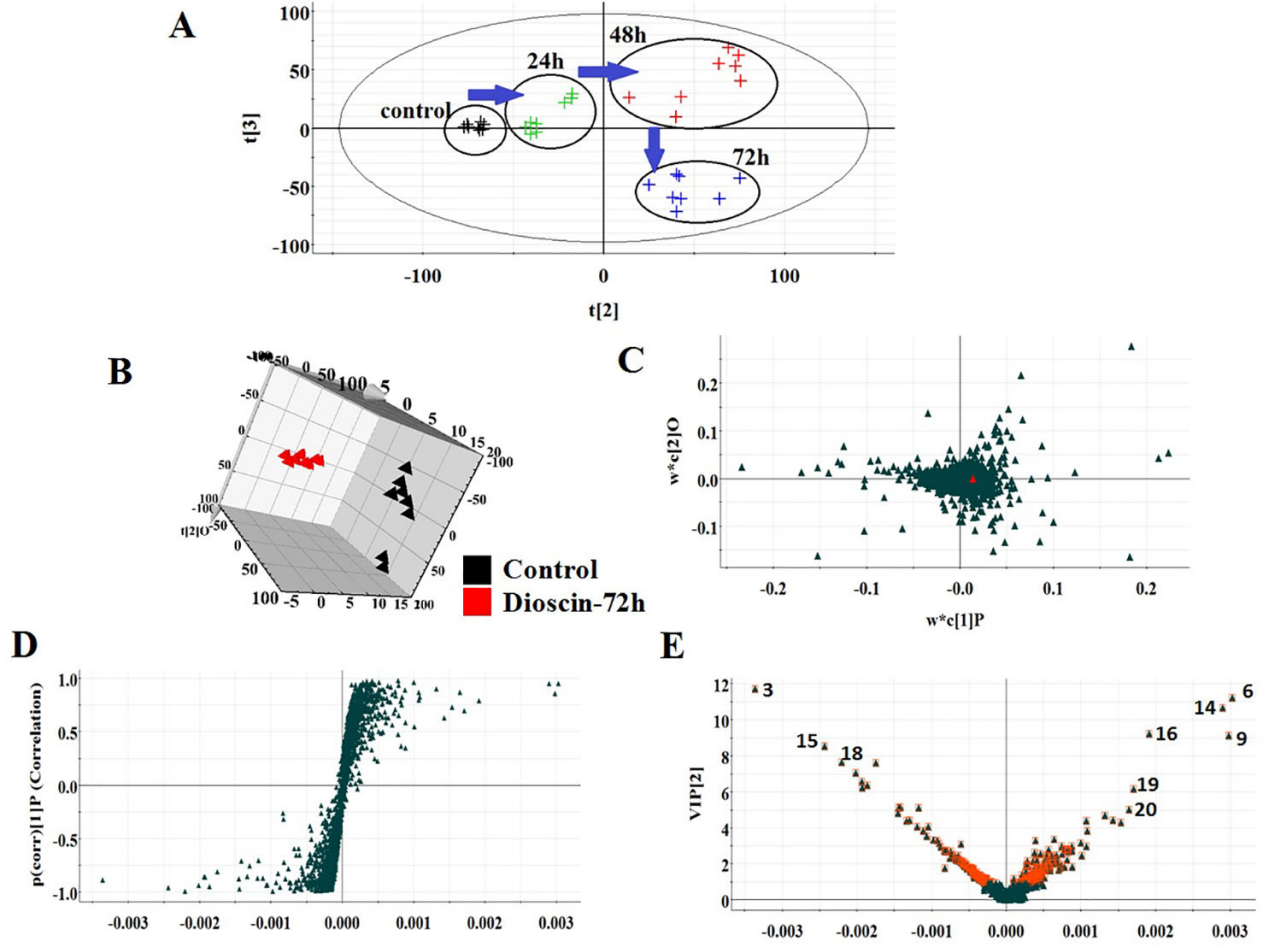
PCA scores’ plot of cell metabolism trajectory in the dioscin-treated group at different time points in negative mode **(A)**. 3D OPLS-DA score plot of cell UPLC-MS spectra data between control and 72 h dioscin-treated group in negative mode **(B)**. Loading-plot of OPLS-DA model of UPLC-MS spectra data between control and 72 h dioscin-treated group in negative mode **(C)**. S-plot of OPLS-DA model of UPLC-MS spectra data between control and 72 h dioscin-treated group in negative mode **(D)**. VIP-plot of OPLS-DA model of UPLC-MS spectra data between control and 72 h dioscin-treated group in negative mode **(E)**.

All the data were resolved in both ion modes using high-throughput metabolomics based on LC-MS conditions described above. Raw data of cell samples were imported into Progenesis QI software for multiple statistical analyses. In **Figures 1A** and **2A**, the PCA score plot of the metabolic route in dioscin-treated sample was clearly discreted, which was distant from the control group orientation, and the bias was the highest at 72 h. Then, a wide multidimensional array including RT, m/z, and peak height intensity between the control group and 72 h dioscin group was taken into EZinfo plug-ins for pattern recognition analysis. In both ion modes (**Figure 1B** and **Figure 2B**), OPLS-DA score plots between the control and 72 h treated groups were well separated suggesting that obvious alteration of fluid in the cell had appeared on the metabolic lay after dioscin treatment. The ions farther away from the origin have the greater the contribution for the change of the metabolic profile trajectory in the loading plot (**Figures 1C** and **2C**) and S-plot (**Figures 1D** and **2D**). With the intention of recognizing and seeking potential biomarker closely related with dioscin activity in positive and negative ion modes (**Figures 1E** and **2E**), the VIP score plot presents the donation of the factors to bunch and prejudice.

### Differentiated Cell Biomarker Recognition

**Figure 3:**
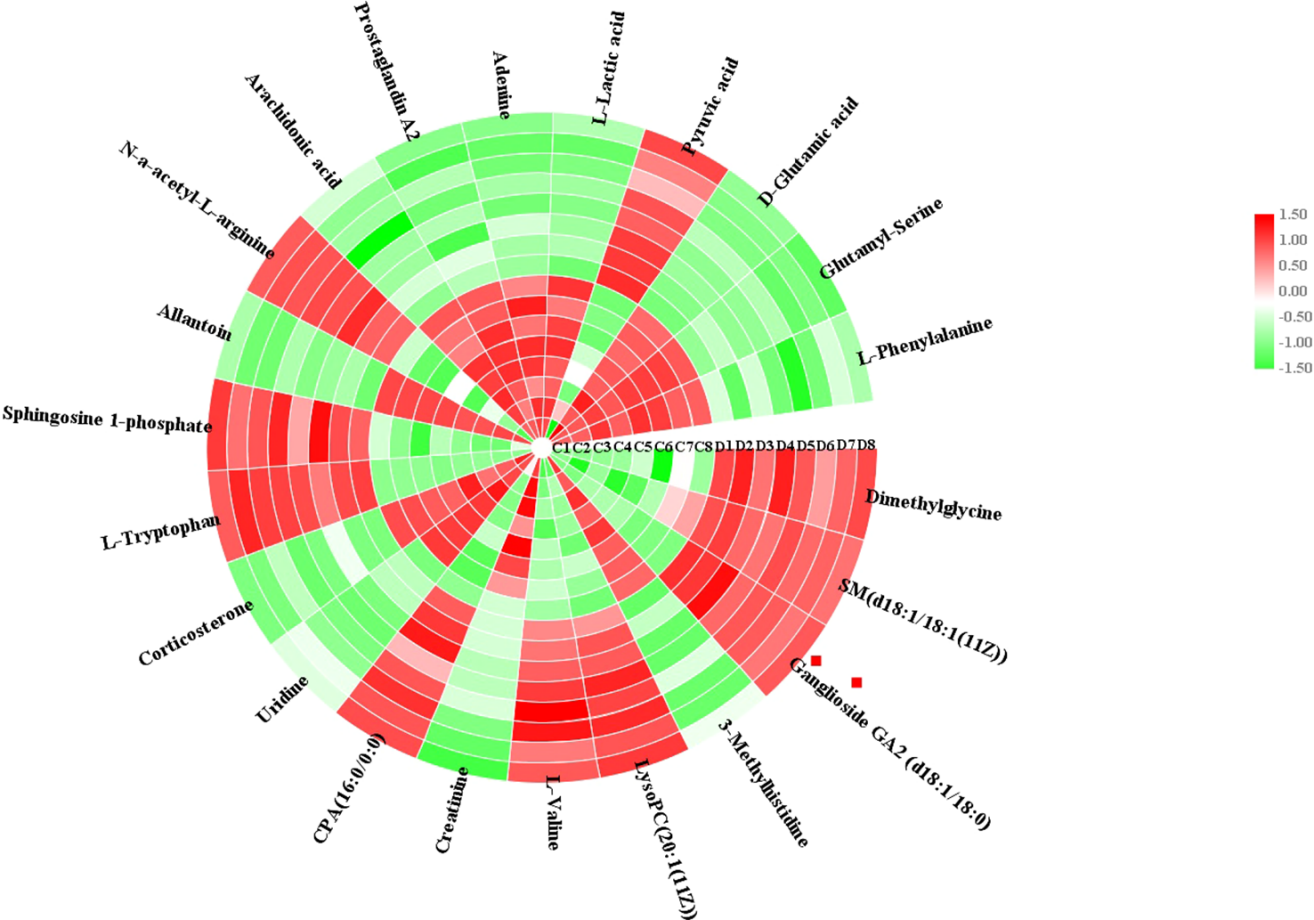
Heatmap visualization of cell biomarker candidates between control and 72 h dioscin administration groups to show significant changes.

**Figure 4:**
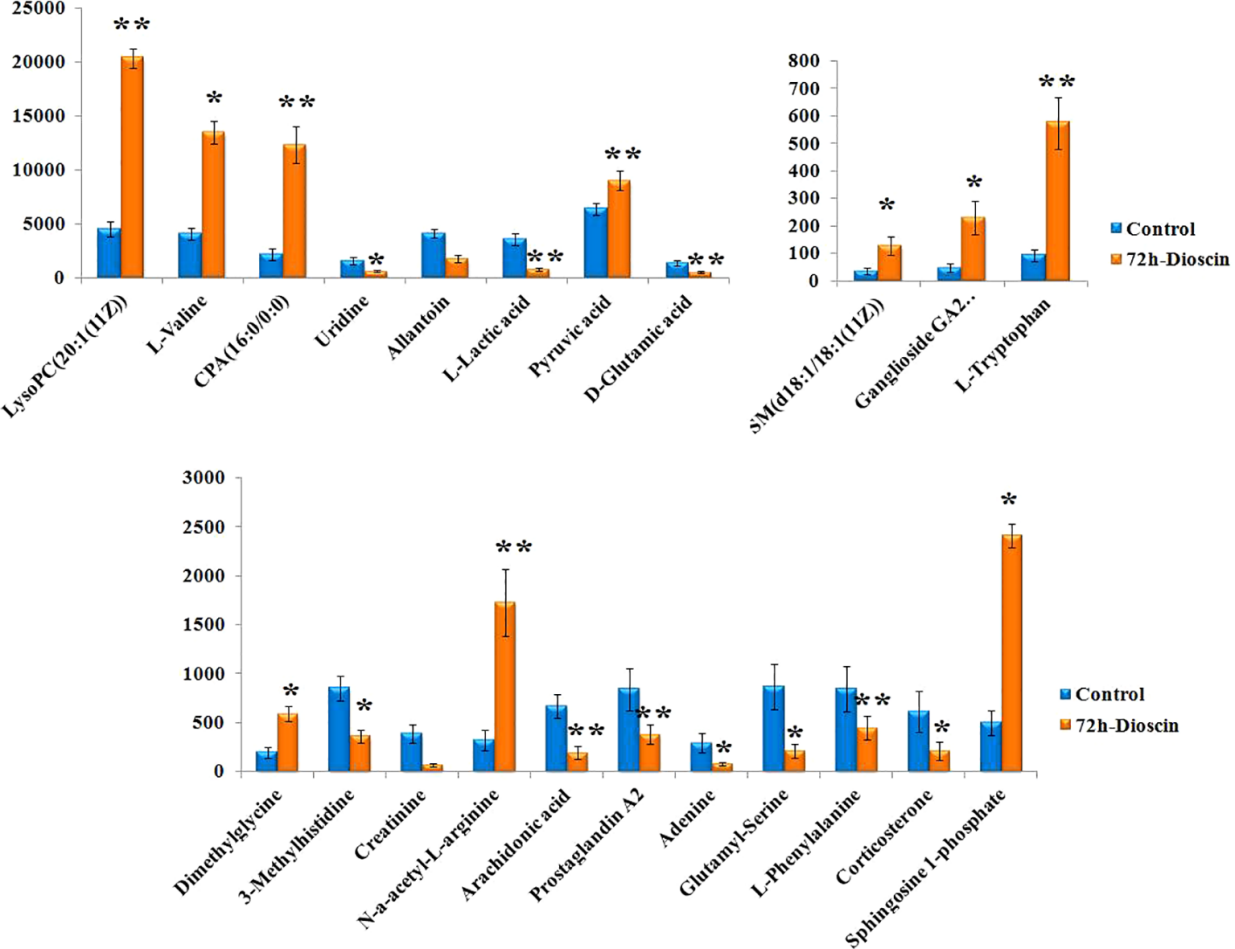
Relative signal intensities for metabolic biomarkers in the cell identified in control and 72 h dioscin administration groups. Compared with control group: *P < 0.05, **P < 0.01.

Using an independent t-test, the metabolites with VIP values were picked from the OPLS-DA which means that P values less than 0.05 were singled out conducing to the clustering differentiation between different groups as potential biomarkers. MS spectrum and relevant MS/MS fragments were used for preliminary analysis, then further, retrieving from online database and searching reference substance were essential to affirm their structure and information. A total of 22 different metabolites were identified in **Table S1**. As shown in **Figure 3**, color heatmap indicated that 10 of the 22 biomarkers were upregulated and 12 of these were downregulated after treatment. Comparative indication strength of 22 cell metabolites from UPLC-MS were calculated and listed in **Figure 4**. Compared with the control group, 10 potential biomarkers, including LysoPC(20:1(11Z)), CPA(16:0/0:0), L-tryptophan, N-a-acetyl-L-arginine, arachidonic acid, prostaglandin A2, L-lactic acid, pyruvic acid, D-glutamic acid, L-phenylalanine, have significant differences for emphasizing the protective effect of dioscin.

### Metabolic Pathways and Network Establishment

**Figure 5:**
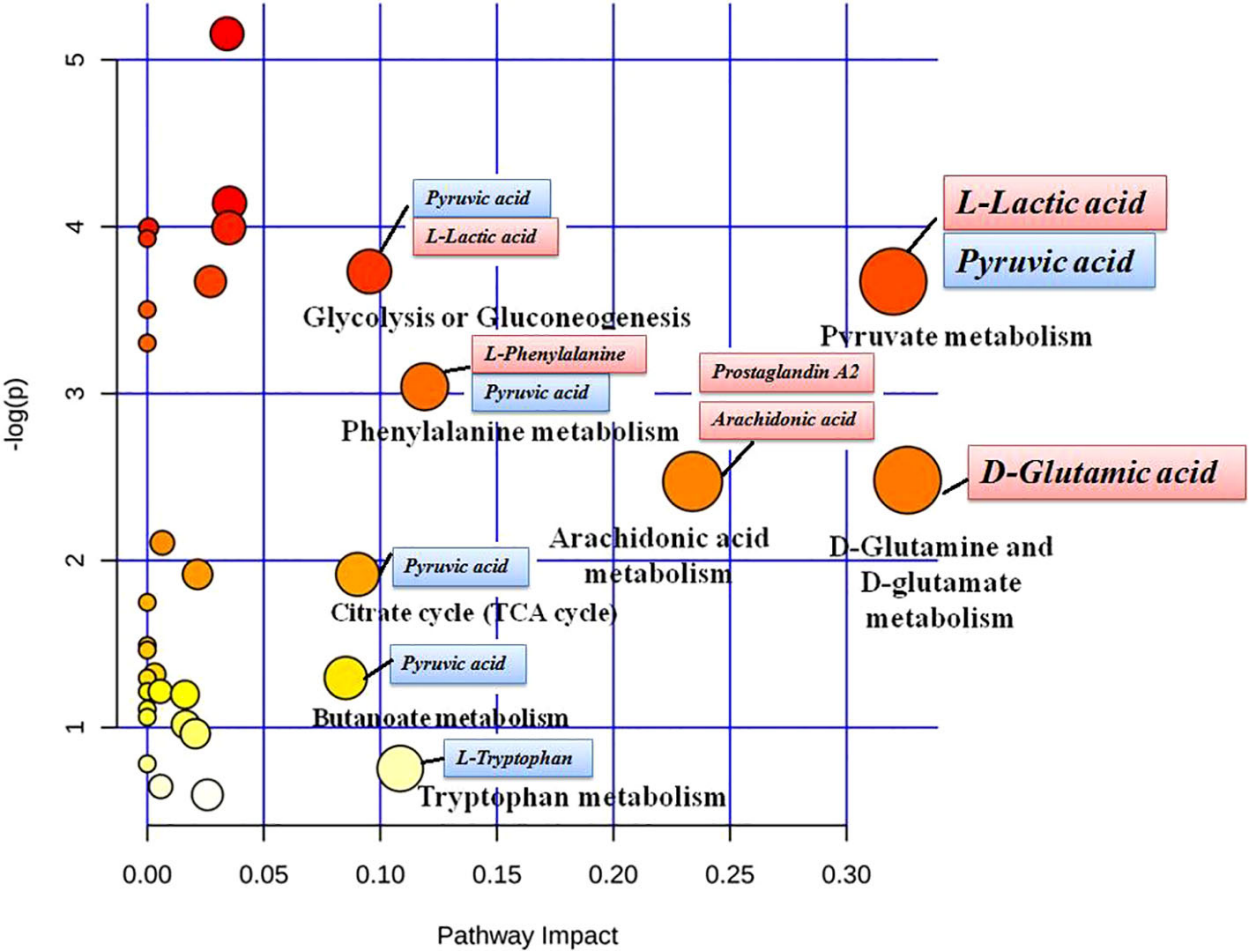
Pathway analysis of metabolic variations after dioscin treatment in cell. The ordinate is the original P values obtained from the pathway analysis, and the abscissa is the influence value of pathways obtained from the topological analysis. They are represented by the depth and size of the color of the circle.

**Figure 6:**
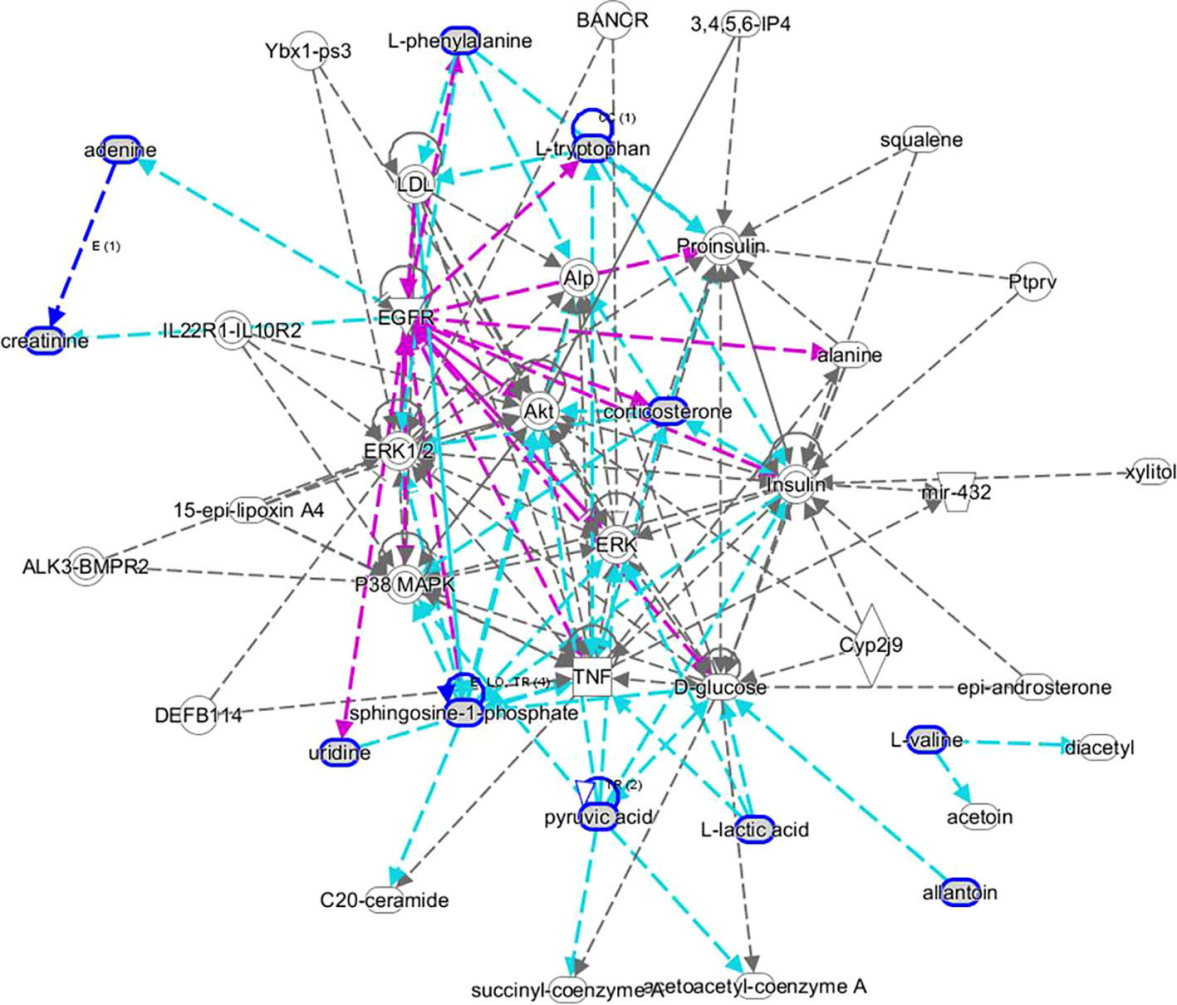
IPA prediction networks associated with dioscin protective activity on SW480 rectal cancer cells.

MetaboAnalyst 4.0 (http://www.metaboanalyst.ca/) was utilized to perform pathway topology analysis and network establishment related to the conditions being studied. It was shown that metabolites regulated by dioscin on rectal cancer were involved in eight key metabolic pathways as follows: D-glutamine and D-glutamate metabolism, pyruvate metabolism, arachidonic acid metabolism, phenylalanine metabolism, tryptophan metabolism, glycolysis or gluconeogenesis, citrate cycle (TCA cycle), and butanoate metabolism (**Figure 5**). As shown in the KEGG global metabolic network, the distributional range of metabolites and enzymes/KEGG Orthologs referred to amino acid metabolism, sphingolipid metabolism, aminoacyl-tRNA biosynthesis, pantothenate, and CoA biosynthesis, glycolysis or Gluconeogenesis, pyruvate metabolism, vitamin B6 metabolism, propanoate metabolism, nitrogen metabolism is depicted (**Figure S3A**). Metabolite–metabolite interaction network highlights the potential functional relationships such as arachidonic acid, pyruvic acid, D-glutamic acid, L-phenylalanine, L-tryptophan, and adenine, which possess similar chemical structures leading to similar molecular activities (**Figure S3B**). In addition, the HMDB information of these 22 metabolites was imported into IPA software for establishing merging metabolic networks to illuminate molecular mechanism of dioscin on SW480 rectal cancer cell, which was concerned with molecule transport, nucleic acid metabolism, molecule biochemistry, cell signaling and interplay, cellular growth and proliferation, cellular compromise (**Figure 6**).

## Discussion

Cancer caused by a combination of various factors is characterized by uncontrolled growth and spread of abnormal cells or tissues, which is next only to cardiovascular disease. It is reported that more than 16 million new cancer cases by 2020 and 10 million people will face the threat of death (Liu et al., 2009). To date, about 60% of anticancer agents and 75% of anti-infectious agents are derived from natural products, and more than 30 natural-derived compounds are used in clinical treatment of various stages of cancer (Das and Satyalakshmi, 2012). The most studied of the natural products include taxol, camptothecin, curcumin, betulinic acid, podophyllotoxin, and resveratrol.

Dioscin is an anticancer natural product in recent years (Brahmbhatt et al., 2015; Vassilakopoulou et al., 2015; Si et al., 2016). Glucose is firstly decomposed into pyruvic acid without aerobic action, and then a tricarboxylic acid (TCA) cycle is performed in the mitochondria to produce a large amount of ATP. However, in the 1950s, Warburg (Sun et al., 2018b) reported that pyruvate decomposed by glucose in tumor cells is converted to lactic acid in the presence of oxygen rather than in the mitochondria. Tumor cells use the metabolic pathways of glucose and its derivatives to produce the green medium needed for their biosynthesis, maintain a good redox circumstance, and meet their required vitality. Pyruvate dehydrogenase (PDH) is a key regulator of pyruvate, which varies pyruvate to acetyl-CoA in the mitochondria. As a vital conditioner of PDH activity is PDH kinase (PDK), it promotes the inactivation of PDH by promoting phosphorylation of specific sites in serine (Zhang et al., 2018), resulting in a decrease in the amount of pyruvate entering the mitochondria and an increase in the amount of lactic acid. Compared with the control group, the level of L-lactic acid is downregulated and the content of pyruvic acid is upregulated after dioscin treatment, which indicated that dioscin monitors pyruvate metabolism for inhibiting abnormal conversion among sugars, proteins, and lipids. In addition to altering glucose metabolism, tumor cells also increase the application and dependence of glutamine to facilitate cell growth and survival. The dramatic increase in glutamine is closely associated with the metabolism of oncogene c-Myc signaling in tumors (Gao et al., 2009) and other oncogene variants, such as K-ras (Son et al., 2013). In the cytosol, glutamine can be employed as a substrate for *de novo* synthesis of proteins, purines, and pyrimidines and can be varied to glutamic acid by glutaminases (GLS). Tumor cells utilize glutamine-derived glutamic acid to perform various activities (Hensley et al., 2013).

As shown in the above result, the level of D-glutamic acid is increased in the control group suggesting that abnormal activity in the pathogenic period of tumor cells has been employed to maintain growth and survival, and then dioscin validly reverses D-glutamine and D-glutamate metabolism. Phenylalanine is one of the eight essential amino acids in the human body, which referred to the formation of various protein components but cannot be synthesized in the human body. Under normal circumstances, about 50% of the ingested phenylalanine is used to synthesize various components of the protein, and the rest is changed to tyrosine by the action of phenylalanine hydroxylase and then converted to dopa, dopamine, adrenaline, norepinephrine and melanin by other enzymes. Phenylalanine hydroxylase is a complex enzyme system including dihydropterin reductase and coenzyme tetrahydrobiopterin. An enzyme deficiency can induce an increase in blood phenylalanine. In the control group, the content of phenylalanine was increased, and dioscin regulated them close to the abnormal level. Activated arachidonic acid (AA) metabolic enzymes and their products arrange the inflammatory reply and adjust multiple cellular procedures such as cell proliferation, survival, penetration, and pervasion, which accelerates carcinogenesis and plays a significant role in inflammation and tumorigenesis. Some studies have suggested that natural as well as synthetical molecules lead to pharmacological prophylaxis of the AA pathway, which stimulates researchers to probe into therapeutic intervention of the AA pathway (Wang and Dubois, 2010; Li et al., 2010). As a cyclopentenone compound and an endogenous metabolite of arachidonic acid, prostaglandin A has ability to resist cell proliferation *in vivo* and *in vitro* (Zhang et al., 2014d). The upregulated level of AA and prostaglandin A is attributed to self-protection of SW480 rectal cancer cells in the inflammatory response. Dioscin exhibit reverses the content of AA and prostaglandin a mediated in AA metabolism.

Metabolomics approach plays a key role in researching the metabolic disorder-related diseases (Zhang et al., 2013d; Zhang et al., 2014e; Zhang et al., 2015b; Sun et al., 2015; Wang et al., 2016; Zhao et al., 2016; Chu et al., 2016; Song et al., 2017; Ren et al., 2018; Qiu et al., 2020), discovery and identification of biomarkers (Zhang et al., 2014f) and treatment (Wang et al., 2012; Liang et al., 2015; Zhang et al., 2016b; Zhao et al., 2017; Liu et al., 2018; Sun et al., 2019; Wu et al., 2019; Qiu et al., 2020). Many biomarkers are being discovered for better understanding of metabolic mechanisms (Ma et al., 2016; Wang et al., 2016; Li et al., 2018) and evaluating the efficacy diseases (Chu et al., 2015a; Chu et al., 2015b; Wang et al., 2016; Zhang et al., 2017). These emerging candidate biomarkers own a promising future, and numerous studies are indispensable to validation of diseases. Our study showed that strategies based on cell metabolomics are helpful tools to discover the small molecular metabolites to elucidate the action mechanism of drug.

## Conclusion

In this study, we used cell metabolomics strategy to explore the effect of dioscin on rectal cancer cells and to discover the small molecular metabolites for elucidating the action mechanism of dioscin. A total of twenty-two differentiated biomarkers were identified with distinct changes in rectal cancer cells. The metabolic mechanism of dioscin on SW480 rectal cancer cells was related to D-glutamine and D-glutamate metabolism, pyruvate metabolism, and arachidonic acid metabolism, respectively. It was implied that cell metabolomics is a beneficial tool to further promote the development of dioscin as antitumor drugs.

## Data Availability Statement

All datasets generated for this study are included in the article/**Supplementary Material**.

## Author Contributions

The list of authors who contributed to this work is as follows: X-XW conceived and designed the experiments. X-XW, P-CY, and JL performed the experiment. X-XW analyzed the data. JL guided the experiment. X-XW wrote the paper. P-CY revised the paper. All authors read and approved the final manuscript.

## Conflict of Interest

The authors declare that the research was conducted in the absence of any commercial or financial relationships that could be construed as a potential conflict of interest.

The reviewer XW declared a shared affiliation, with no collaboration, with one of the authors JL to the handling editor at time of review.
