# Supplementary material for: High-Throughput Metabolomics for Identification of Metabolic Pathways and Deciphering the Effect Mechanism of Dioscin on Rectal Cancer From Cell Metabolic Profiles Coupled With Chemometrics Analysis
Source: Front Pharmacol. 2020 Feb 28;11:68. doi: 10.3389/fphar.2020.00068 (PMC7059176; doi:10.3389/fphar.2020.00068)
Supplement: Supplementary file 1 [file DataSheet_1.doc]

**Table S1** Differentiated metabolites identified in rectal cancer cells by LC- MS in the positive and negative ion mode.

|  | **Rt(min)** | **Ion form** | **Formula** | **Proposed compound** | **m/z** | **HMDB** |
| --- | --- | --- | --- | --- | --- | --- |
| 1 | 0.21 | M+H | C4H9NO2 | Dimethylglycine | 104.07 | HMDB00092 |
| 2 | 7.95 | M+H | C41H81N2O6P | SM(d18:1/18:1(11Z)) | 729.59 | HMDB12100 |
| 3 | 5.97 | M-H | C56H104N2O18 | Ganglioside GA2- (d18:1/18:0) | 1091.73 | HMDB04891 |
| 4 | 3.70 | M+H | C7H11N3O2 | 3-Methylhistidine | 192.07 | HMDB00479 |
| 5 | 6.30 | M+H | C28H56NO7P | LysoPC(20:1(11Z)) | 550.39 | HMDB10391 |
| 6 | 0.63 | M-H | C5H11NO2 | L-Valine | 117.15 | HMDB00883 |
| 7 | 0.65 | M+H | C4H7N3O | Creatinine | 114.07 | HMDB00562 |
| 8 | 4.83 | M+H | C19H37O6P | CPA(16:0/0:0) | 415.22 | HMDB07003 |
| 9 | 1.53 | M-H | C9H12N2O6 | Uridine | 243.06 | HMDB0000296 |
| 10 | 2.56 | M+H | C21H30O4 | Corticosterone | 347.22 | HMDB0001547 |
| 11 | 2.39 | M+H | C11H12N2O2 | L-Tryptophan | 205.09 | HMDB0000929 |
| 12 | 9.58 | M+H | C18H38NO5P | Sphingosine 1-phosphate | 380.26 | HMDB00277 |
| 13 | 0.66 | M+H | C4H6N4O3 | Allantoin | 158.12 | HMDB00462 |
| 14 | 0.90 | M-H | C8H16N4O3 | N-a-acetyl-L-arginine | 216.24 | HMDB04620 |
| 15 | 3.98 | M-H | C20H32O2 | Arachidonic acid | 303.23 | HMDB01043 |
| 16 | 5.04 | M-H | C20H30O4 | Prostaglandin A2 | 333.20 | HMDB02752 |
| 17 | 1.73 | M+H | C5H5N5 | Adenine | 136.06 | HMDB00034 |
| 18 | 8.30 | M-H | C3H6O3 | L-Lactic acid | 89.02 | HMDB00190 |
| 19 | 0.55 | M-H | C3H4O3 | Pyruvic acid | 87.01 | HMDB00243 |
| 20 | 0.52 | M-H | C5H9NO4 | D-Glutamic acid | 146.05 | HMDB03339 |
| 21 | 0.53 | M+H | C8H13N2O6 | Glutamyl-Serine | 234.08 | HMDB28828 |
| 22 | 2.24 | M+H | C9H11NO2 | L-Phenylalanine | 166.09 | HMDB00159 |


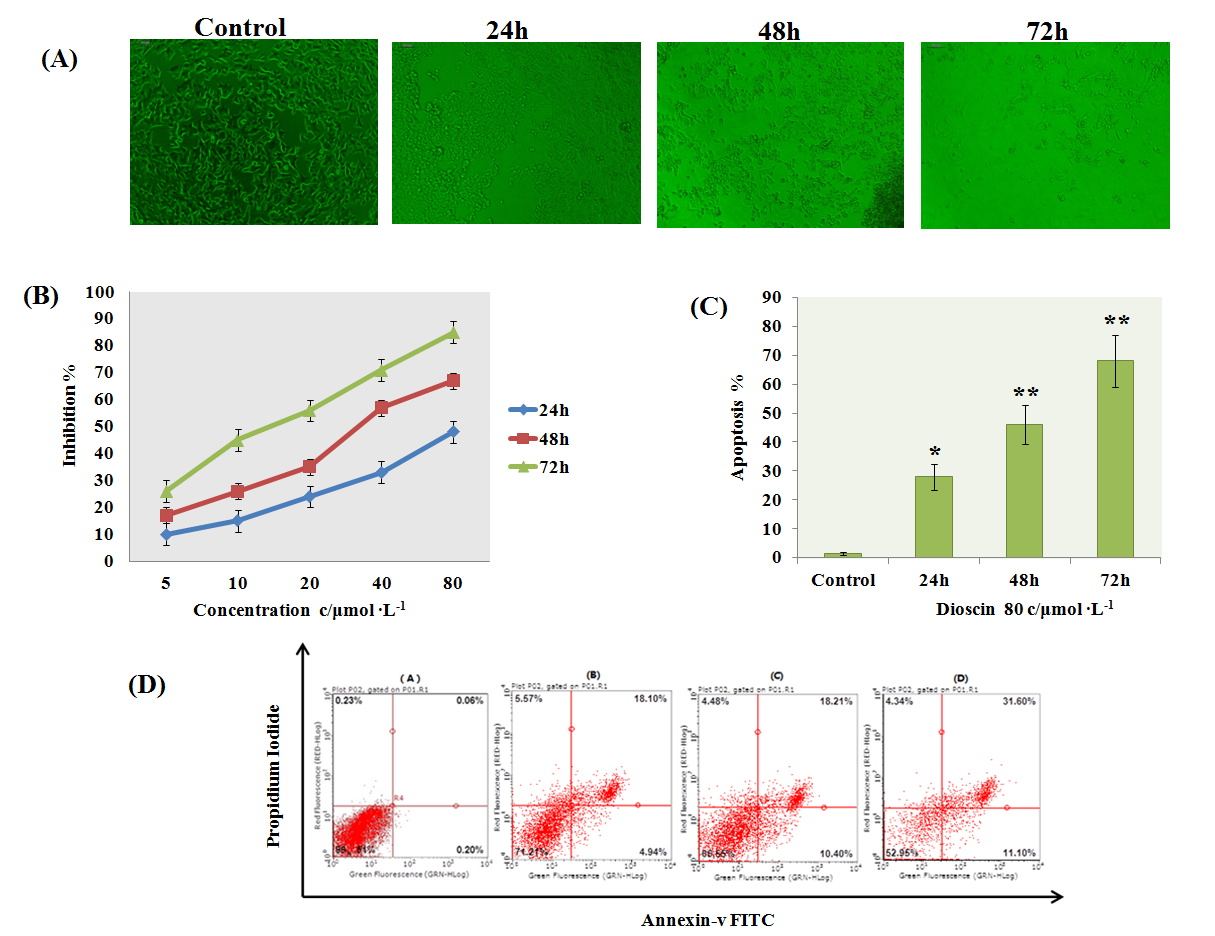


Figure S1 (A) Cell morphological changes of SW480 rectal cancer cells caused by Dioscin treatment at different time. (B) Dioscin inhibited the proliferation of SW480 rectal cancer cells at the concentrations of 5 c/μmol·L^-1^, 10 c/μmol·L^-1^, 20 c/μmol·L^-1^, 40 c/μmol·L^-1^, 80 c/μmol·L^-1^ for 24 h, 48 h, 72 h. (C) and（D） Dioscin induce the apoptosis rate change and annexin V-FITC/PI staining analysis of SW480 rectal cancer cells for 24 h, 48 h, 72 h at the concentrations of 80 c/μmol·L^-1^. * P < 0.05, ** P < 0.01


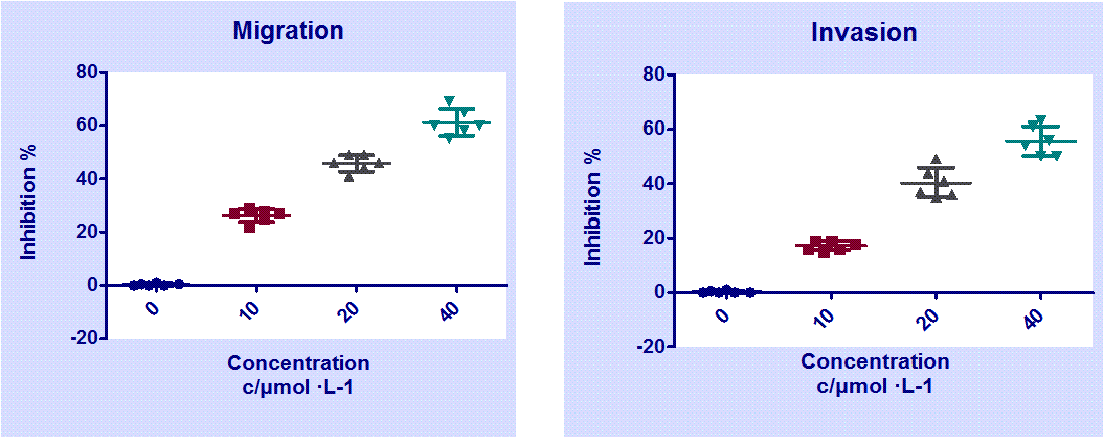


Figure S2 The analysis of migration and invasion inhibition changes of SW480 rectal cancer cells by Dioscin treatment at the concentrations of 10 c/μmol·L^-1^, 20 c/μmol·L^-1^, 40 c/μmol·L^-1^.

**
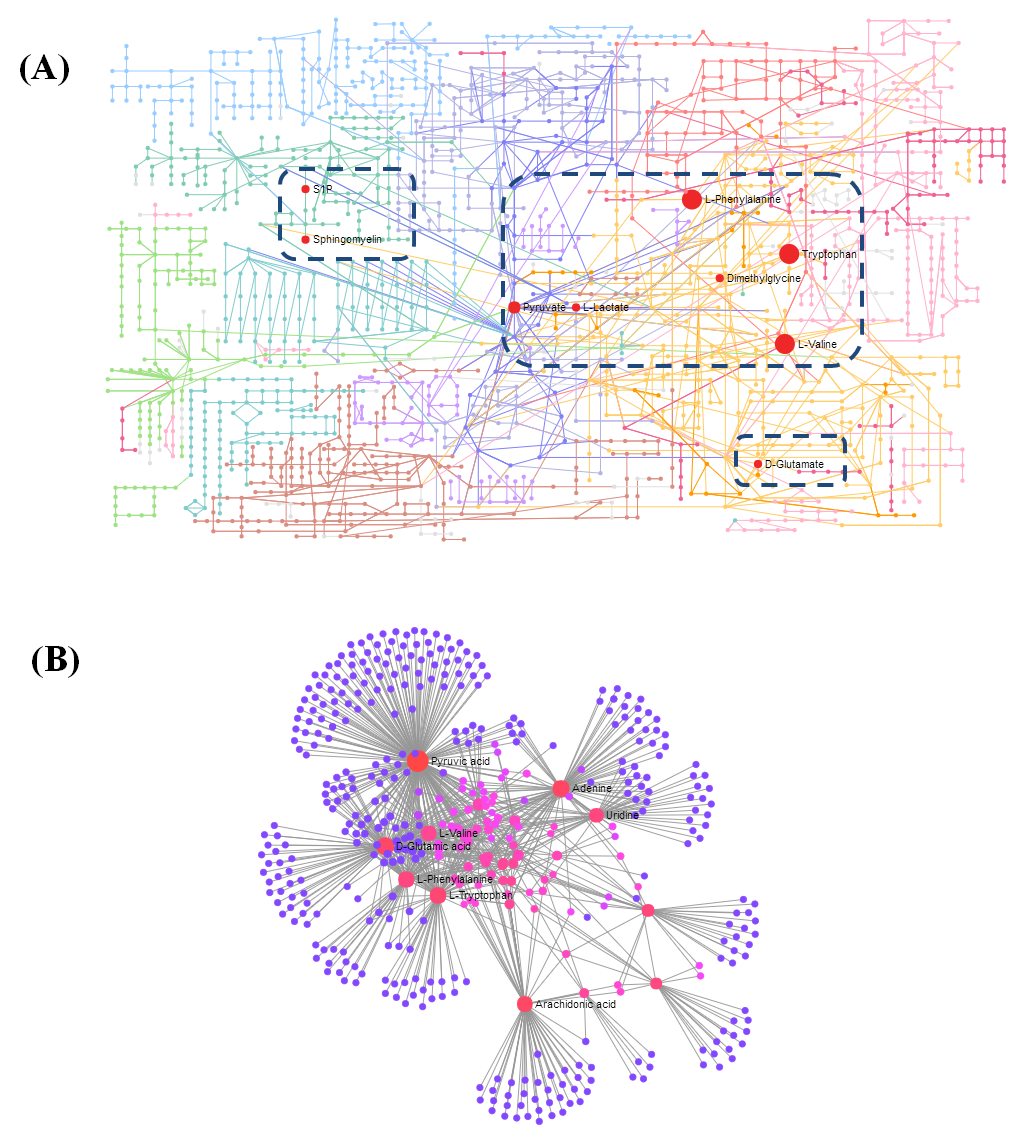
**

Figure S3 KEGG global metabolic network related with the protective activity of Dioscin on SW480 rectal cancer cells (A), including glycine, serine and threonine metabolism; sphingolipid metabolism; aminoacyl-tRNA biosynthesis; valine, leucine and isoleucine biosynthesis; phenylalanine, tyrosine and tryptophan biosynthesis; pantothenate and CoA biosynthesis; glycolysis or gluconeogenesis;pyruvate metabolism; vitamin B6 metabolism; propanoate metabolism; nitrogen metabolism; phenylalanine metabolism. Metabolite-metabolite interaction network related with the protective activity of Dioscin on SW480 rectal cancer cells (B), which refers to arachidonic acid; pyruvic acid; D-glutamic acid; L-phenylalanine; L-tryptophan; adenine; L-valine; uridine .
